# Obesity management: Attitudes and practice of Italian endocrinologists

**DOI:** 10.3389/fendo.2022.1061511

**Published:** 2023-01-17

**Authors:** Marco Chianelli, Luca Busetto, Roberto Attanasio, Olga Eugenia Disoteo, Giorgio Borretta, Agnese Persichetti, Irene Samperi, Alessandro Scoppola, Agostino Paoletta, Franco Grimaldi, Enrico Papini, Antonio Nicolucci

**Affiliations:** ^1^ Endocrinology Unit, Regina Apostolorum Hospital, Rome, Italy; ^2^ Endocrinology and Metabolism - Regina Apostolorum Hospital, Roma, Italy; ^3^ Department of Medicine, University of Padova, Padova, Italy; ^4^ Scientific Committee of the Italian Association of Clinical Endocrinologists, Milan, Italy; ^5^ SC Diabetologia, ASST Grande Ospedale Metropolitano Niguarda, Milan, Italy; ^6^ Department of Endocrinology, Diabetes and Metabolism, Santa Croce & Carle Hospital, Cuneo, Italy; ^7^ Service of Pharmacovigilance, IRCCS-Regina Elena National Cancer Institute, Rome, Italy; ^8^ SSD of Diabetology, Azienda Sanitaria Locale, Novara, Italy; ^9^ Endocrinology Unit, Santo Spirito Hospital, Rome, Italy; ^10^ Endocrinology, ULSS 6 Euganea, Padova, Italy; ^11^ Endocrinology, Diseases of Metabolism and Clinical Nutrition Unit, University Hospital S.M. Misericordia, Udine, Italy; ^12^ Center for Outcomes Research and Clinical Epidemiology - CORESEARCH, Pescara, Italy

**Keywords:** obesity, endocrinologists, attitudes, survey, treatment

## Abstract

**Introduction:**

Obesity is a global pandemic and is cause of serious concern in all regions of the world. It is important to raise the attention of health care professionals in order to provide early treatment of patients with obesity. Obesity management, however, varies greatly amongst endocrinologists with respect to attitudes to diagnosis and treatment. Aim of this study was to identify practices and needs of Italian endocrinologists with respect to people with obesity.

**Methods:**

In this study, all members of the Italian Association of Clinical Endocrinologists (AME) were invited to participate in a web-based survey concerning the management of obesity.

**Results:**

The response rate was 24.1% (542/2248). Nutritional and obesity problems were reported as major areas of interest by 29.4% of the participants. A large proportion of patients seeking an endocrine consultation for other reasons are affected by obesity, but one in five respondents addressed the issue in 25% or less of the cases, while one in three always dealt with the problem. Obesity was managed personally/within a dedicated team by 42.6% of participants, while the remainders referred the patient to a dietician/nutritionist or a 2nd level center for obesity therapy. Metformin was used in a median of 30% of the patients (Interquartile range: 10-50) and liraglutide in 10% of the cases (IQR 0-30), while orlistat (median 0%; IQR 0-10) and naltrexone/bupropion (median 0%; IQR 0-5) were seldom prescribed. Cost of therapy was considered as the major limitation to the use of anti-obesity drugs, affecting adherence to long-term treatment. According to 41.9% of respondents, psychological support should be offered to all patients with obesity. Finally, 56% of participants believe that the availability of new drugs will increase the number of patients candidate to drug therapy.

**Discussion:**

In conclusion, it is of primary importance to raise the awareness of endocrinologists towards the problem of obesity and increase their confidence in managing this pathological condition.

## Introduction

The continuing rise in the prevalence of overweight and obesity is increasingly taking the form of a global pandemic, and is a cause of serious concern in all regions of the world. In 2015, a total of 107.7 million children and 603.7 million adults had obesity (1). The high body mass index (BMI) was responsible, directly or indirectly, for 4.0 million deaths globally, and more than two thirds of high BMI-related deaths were due to cardiovascular disease (1).

In Europe, the prevalence of obesity has tripled in many countries since the 1980s and continues to grow at an alarming rate, especially among children. According to recent WHO estimates, one in two citizens in Europe have overweight or obesity, while one in five suffers from obesity (2). In Italy, data from the National Institute of Statistics document between 2001 and 2010 a growth of about two million in the number of people with overweight and over one million for people with obesity. In 2016 in Italy, over 23 million adults (45.9% of the population) had overweight or obesity (17.9 million with overweight and 5.2 million with obesity) (3).

Excess weight is responsible for a high clinical, social and economic burden, related to the multitude of associated pathological conditions, such as diabetes, cardiovascular diseases, respiratory diseases, some types of cancer and osteoarticular diseases (4). Furthermore, people living with obesity experience stigma, discrimination and a negative impact on both mental and health-related quality of life (5). For this reason, obesity warrants recognition by health-care providers and payers, and must be considered as a chronic disorder that requires continuous care, support, and follow-up (6).

Treatment guidelines for people with obesity typically recommend lifestyle interventions, followed by pharmacotherapy if response to dietary, physical activity and behavioral changes alone is insufficient to reach or maintain the recommended goal of 5%–10% loss in body weight (7–10). Bariatric surgery should be considered for severe cases (BMI ≥40 kg/m^2^ or ≥35 kg/m^2^ with obesity-related complications) and for individuals with BMI ≥30 kg/m^2^ and poorly controlled type 2 diabetes.

Despite the availability of evidence-based guidelines (7–10), the increasing prevalence of obesity suggests that these recommendations are poorly implemented. Suboptimal care to people with obesity has been frequently described (11–13), along with low rates of obesity diagnosis, documentation and management (14, 15), and inadequate knowledge of obesity treatment guidelines (16). Furthermore, few people with obesity receive weight-loss counselling, and of those, approximately one quarter have a follow-up appointment scheduled to review their weight (14, 16). Studies also show a wide variation in the provision of pharmacological and/or surgical interventions by healthcare professionals (14, 17), suggesting a lack of familiarity with indications for initiating treatment and referrals and misperceptions of the safety and/or efficacy of currently available weight loss medications and bariatric surgery (13, 18).

Clinical endocrinologists can represent an important resource to identify, evaluate and manage obesity, in the light of the high percentage of patients attending their offices showing excess body weight. Aim of this study was to assess practices and attitudes of Italian endocrinologists regarding care of people with obesity.

## Materials and methods

This was a cross-sectional survey study of endocrinologists, members of the Italian Association of Clinical Endocrinologists (AME). Participants were asked to complete an online multiple-choice questionnaire that was kept open for 4 weeks. We utilized a web-based survey constructed with Lime-Survey, an open-access platform that provides various question templates. The survey link was sent *via* email to 2248 endocrinologists, followed by weekly reminders. Participation was voluntary without compensation, and responses were anonymous. Survey responses were collected and electronically stored by the survey service, which were accessible by password. The survey service automatically blocked repeat submissions from the same IP address.

The questionnaire aimed to assess (1) how often endocrinologists address obesity (patients with a BMI ≥30 kg/m^2^), (2) how obesity is managed, (3) the first approach usually adopted in patients with various degrees of obesity, (4) beliefs regarding ketogenic diet and substitute foods, (5) kind of physical activity prescribed to people with obesity (6) attitudes to recommend psychological support in the management of obesity, (7) frequency of use of anti-obesity medications and the reasons for their choice, (8) major obstacles to prescribing medications for obesity, (9) major factors affecting treatment adherence, (10) awareness of new anti-obesity medications coming on the market and expectations of benefits of new drugs.

The questionnaire also included demographic data of the participant, such as sex, age, years of practice, main areas of practice, kind and setting of practice, availability in the structure of a specialist or unit dedicated to obesity.

### Statistical analysis

Continuous data are summarized as median and interquartile range (IQR), while categorical data are reported as percentages. We examined bivariate association of physician characteristics with responses to selected items using the Kruskal Wallis one-way ANOVA in case of continuous variables and the chi square test in case of categorical variables.

Correlates of attitudes to prescribe the different anti-obesity drugs were investigated using logistic regression analysis with stepwise variable selection. The following respondent characteristics were tested in each model: age, gender, setting of practice, main area of interest, attitude to manage obesity. Results of logistic regression analyses are reported as Odds Ratios (ORs) with their 95% Confidence Interval (95%CI).

P-values <0.05 were considered as statistically significant. Statistical analyses were performed using the SPSS software ver. 23.0 (IBM, Armonk, NY, USA).

## Results

Overall, 542 out of 2248 endocrinologists took part to the survey (24.1%). Participants characteristics are reported in **Table 1**. The sample was well distributed in terms of gender, age classes and years of practice. Main areas of practice were represented by thyroid diseases, general endocrinology and diabetes mellitus. Nutritional and obesity problems were reported as one of the major areas of interest by 29.4% of the participants. Half of the endocrinologists practiced in a public hospital setting, either affiliated (18.0%) or non-affiliated (35.2%) to a university. The remainders practiced in a public (16.7%) or private (24.2%) outpatient clinic. A specialist or unit dedicated to obesity was available in the structure of practice of 52.1% of the participants.

**Table 1:** Characteristics of respondents.

Characteristics	N (%)
Gender	
Male	218 (40.2%)
Female	318 (58.7%)
NR	6 (1.1%)
Age (years)	
<30	115 (21.5%)
30-40	97 (18.2%)
41-50	135 (25.3%)
51-60	125 (23.4%)
61-70	18 (3.4%)
>70	37 (6.9%)
NR	7 (1.3%)
Years of practice	
<5	65 (12.2%)
5-10	72 (13.5%)
11-20	121 (22.7%)
21-30	110 (20.6%)
31-40	111 (20.8%)
>40	35 (6.6%)
NR	20 (3.7%)
Main areas of practice*	
Thyroid	349 (65.4%)
General endocrinology	284 (53.2%)
Diabetes mellitus	253 (47.4%)
Nutrition/obesity	157 (29.4%)
Osteoporosis	90 (16.9%)
Pituitary gland	51 (9.6%)
Oncological endocrinology	42 (7.9%)
Adrenal gland	39 (7.3%)
Mineral metabolism	29 (5.4%)
Andrology	24 (4.5%)
Pediatric endocrinology	16 (3.0%)
Reproduction	12 (2.2%)
Endocrinological surgery	4 (0.7%)
Kind of practice	
Private practice	164 (30.7%)
Hospital	206 (38.6%)
Outpatient clinic	79 (14.8%)
Resident doctor	30 (5.6%)
University	16 (3.0%)
Other/NR	39 (7.3%)
Setting of practice	
Non-University hospital	188 (35.2%)
University hospital	96 (18.0%)
Public outpatient clinic	89 (16.7%)
Private outpatient clinic	129 (24.2%)
Other/NR	32 (6.0%)
Availability in the structure of a specialist or unit dedicated to obesity	
Yes	278 (52.1%)
No	210 (39.3%)
Don’t know	13 (2.4%)
NR	33 (6.2%)

NR, not reported.

* more than one answer was allowed.

### Management of obesity

Answers to the questions included in this section of the survey are reported in **Table 2**. Among the participants, 44.7% reported that 20% to 35% of the patients seen during one month have obesity, while higher percentages were reported by 37.8% of respondents. During the visit of a patient with obesity seeking care for another endocrinological problem, one in five respondents inconstantly (25% or less of the cases) addressed the issue of obesity, while one in three (36.5%) always dealt with the problem. In such circumstances, 42.6% of participants reported they managed obesity personally (29.1%) or within a dedicated team (13.5%), while the remainders tended to refer the patient to a dietician/nutritionist (43.2%), or, less frequently, to a 2^nd^ level center for obesity therapy (6.6%). The attitude to address obesity according to respondents’ characteristics is reported in **Table 3**. Younger endocrinologists and those practicing in a hospital setting were less likely to address obesity in 100% of their patients. On the other hand, participants who managed obesity personally or within a dedicated team, and those reporting obesity/nutrition as a major area of practice were more likely to address obesity in all patients.

**Table 2:** Management of obesity.

	N (%)
How many patients, among those you see in a month, have obesity?	
<20%	87 (17.5%)
20-35%	222 (44.7%)
36-50%	124 (24.9%)
>50%	64 (12.9%)
During the visit of a patient with obesity who came for another endocrinological problem, how often is the problem of obesity addressed?	
never	9 (1.8%)
in 25% of cases	102 (20.7%)
in 50% of cases	111 (22.5%)
in 75% of cases	91 (18.5%)
always	180 (36.5%)
How do you manage obesity in a patient who arrived at your clinic for diseases other than obesity?	
I manage it personally	142 (29.1%)
I manage it in the context of a dedicated team	66 (13.5%)
I refer the patient to a dietician/nutritionist in the center where I work	145 (29.7%)
I refer the patient to a dietician/nutritionist in another center	73 (15.0%)
I refer the patient to a 2nd level center for obesity therapy	32 (6.6%)
I invite the General Practitioner to deal with the problem	7 (1.4%)
Other	23 (4.7%)
Who refers patients with obesity to you? *	
General practitioners	262 (54.1%)
Other specialists	150 (31.0%)
Direct access	201 (41.5%)
Nobody refers to me this kind of patients	82 (16.9%)
What is your first approach to treating patients with BMI between 30 and 34?	
Lifestyle intervention	42 (8.8%)
Psychological/behavioral intervention	11 (2.3%)
Pharmacological	313 (65.2%)
Surgical (only if comorbidities are present)	47 (9.8%)
Nothing	10 (2.1%)
Other	57 (11.9%)
What is your first approach to treating patients with BMI between 35 and 39?	
Lifestyle intervention	15 (3.2%)
Pharmacological	182 (38.5%)
Surgical (only if comorbidities are present)	85 (18.0%)
Surgical	24 (5.1%)
Referral to a 2^nd^ level center	154 (32.6%)
Other	13 (2.7%)
What is your first approach to treating patients with BMI ≥40?	
Lifestyle intervention	3 (0.6%)
Pharmacological	59 (12.6%)
Surgical (only if comorbidities are present)	77 (16.5%)
Surgical	122 (26.1%)
Referral to a 2^nd^ level center	202 (43.3%)
Other	4 (0.9%)
When do you think it is useful to prescribe a ketogenic diet?	
Never	70 (15.2%)
Always	23 (5.0%)
As a first approach after the failure of lifestyle intervention	193 (41.8%)
In case of failure of pharmacological treatment	73 (15.8%)
As bridge therapy before bariatric surgery	103 (22.3%)
What is the usefulness of substitute foods? *	
None	46 (10%)
Complementary	91 (19.7%)
The cost limits their use	165 (35.8%)
It depends on the individual patient	174 (37.7%)
I do not know	56 (12.1%)
What type of exercise do you recommend to your patient with obesity? *	
Anaerobic exercises	17 (3.8%)
Aerobic exercises	208 (45.9%)
Strength exercises	8 (1.8%)
Flexibility exercises	7 (1.5%)
Individualized exercises in relation to the type of patient	216 (47.7%)
The duration and intensity of the exercise are important, not the type	51 (11.3%)
Mixed program that includes all types of exercise	80 (17.7%)
Exercise rarely solves the problem	13 (2.9%)

* more than one answer was allowed.

**Table 3:** Reported percentage of patients with obesity presenting for another problem in whom the obesity problem is addressed, by respondent characteristics.

Respondent characteristics	Percentage of patients					p*
	0%	25%	50%	75%	100%	
Age						0.01
≤40	1.60%	23.40%	24.20%	28.20%	22.60%	
41-60	1.80%	20.00%	20.90%	20.80%	40.50%	
>60	2.00%	19.50%	23.50%	16.80%	42.30%	
Gender						0.5
Male	2.90%	22.50%	22.10%	17.60%	34.80%	
Female	1.00%	19.40%	22.80%	19.00%	37.70%	
Setting of practice						0.002
Non-University hospital	2.70%	20.30%	25.80%	22.50%	28.60%	
University hospital	3.20%	25.50%	27.70%	17.00%	26.60%	
Public outpatient clinic	1.10%	25.30%	14.90%	16.10%	42.50%	
Private outpatient clinic	0.00%	15.00%	18.10%	15.00%	52.00%	
Management of obesity						<0.0001
Personally/within a dedicated team	1.00%	11.50%	17.30%	21.60%	48.60%	
Referral to a dietician/nutritionist	2.30%	21.40%	25.20%	17.00%	27.10%	
Referral to a 2^nd^ level structure	3.10%	31.30%	18.80%	15.60%	31.30%
Main area of interest						0.08
Obesity/nutrition	1.90%	14.90%	16.20%	19.50%	47.40%	
Thyroid	2.20%	21.10%	25.10%	17.50%	34.10%	
Diabetes	0.00%	27.00%	25.40%	20.60%	27.00%	
General endocrinology/other	1.90%	28.30%	26.40%	17.00%	26.40%	

* Chi-square test.

In case of a patient with BMI between 30 and 34 kg/m^2^, the most common approach reported by participants was pharmacological treatment (65.2%), followed by bariatric surgery in case of comorbidities (9.8%), and lifestyle intervention (8.8%) (**Table 2**). In the presence of more severe obesity (BMI between 35 and 39 kg/m^2^), the preferred approach was pharmacological treatment for 38.5% of respondents, followed by referral to a 2^nd^ level center (32.6%), and surgery only if comorbidities are present (18.0%). In the most severe forms of obesity (BMI ≥40 kg/m^2^), endocrinologists tended to refer patients to a 2^nd^ level center (43.3%) or surgery (26.1%).

The vast majority of respondents considered useful to prescribe a ketogenic diet, particularly as a first approach after the failure of lifestyle intervention (41.8%), as bridge therapy before bariatric surgery (22.3%), or in case of failure of pharmacological treatment (15.8%).

The types of exercise most frequently recommended are represented by individualized exercises in relation to the type of patient (47.7%), and aerobic exercises (45.9%).

### Pharmacological treatment of obesity

Answers to the questions included in this section of the survey are reported in **Table 4**. On average, metformin was used in 30% of patients (IQR 10-50). The main reasons for prescribing metformin were represented by the presence of hyperinsulinemia (85.7%) and metabolic complications (41.8%).

**Table 4:** Pharmacological treatment of obesity.

	N (%) or
	median [IQR]
In what percentage of patients do you use metformin?	30 [10-50]
The prescription of metformin is dictated by:*	
BMI	82 (21.3%)
failure of lifestyle changes	75 (19.5%)
hyperinsulinemia	330 (85.7%)
metabolic complications	161 (41.8%)
other complications	8 (2.1%)
failure/intolerance of other drug therapy for obesity	20 (5.2%)
cost	47 (12.2%)
In what percentage of patients do you use orlistat?	0 [0-10]
The prescription of orlistat is dictated by:*	
BMI	75 (36.9%)
failure of lifestyle changes	118 (58.1%)
metabolic complications	42 (20.7%)
other complications	7 (3.4%)
failure/intolerance of other drug therapy for obesity	75 (36.9%)
cost	10 (4.9%)
In what percentage of patients do you use naltrexone/bupropion?	0 [0-5]
The prescription of naltrexone/bupropion is dictated by:*	
BMI	64 (40.8%)
failure of lifestyle changes	93 (59.2%)
metabolic complications	29 (18.5%)
other complications	16 (10.2%)
failure/intolerance of other drug therapy for obesity	77 (49.0%)
cost	14 (8.9%)
In what percentage of patients do you use liraglutide?	10 [0-30]
The prescription of liraglutide is dictated by:*	
BMI	179 (57.4%)
failure of lifestyle changes	183 (58.7%)
metabolic complications	210 (67.3%)
other complications	48 (15.4%)
failure/intolerance of other drug therapy for obesity	85 (27.2%)
cost	27 (8.7%)
What is the major obstacle to prescribing drug therapy for obesity? *	
Side effects	150 (34.6%)
Cost	312 (71.9%)
Resistance on the part of the patient	95 (21.9%)
Poor effectiveness	63 (14.5%)
Limited durability	111 (25.6%)
Distrust on the part of the prescriber	28 (6.5%)
In what percentage of cases do you think obesity can be successfully managed in the long term with lifestyle intervention combined with drug therapy?	
<10%	58 (13.4%)
10-30%	164 (38.0%)
31-50%	123 (28.5%)
>50%	87 (20.1%)
Who do you think should be recommended psychological support in the management of obesity?	
everyone	181 (41.9%)
over half of the cases	139 (32.2%)
25-50% of cases	69 (16%)
<25% of cases	30 (6.9%)
only in the presence of psychiatric problems	13 (3.0%)
What factors negatively affect adherence and therefore the persistence of the results of drug therapy?*	
Side effects	193 (44.9%)
Cost	279 (64.9%)
Insufficient motivation	238 (55.3%)
Therapeutic failure	164 (38.1%)
Distrust of the prescriber	28 (6.5%)
Are you aware of new drugs coming for obesity therapy?	
Yes	173 (40.5%)
No	254 (59.5%)
Do you think the availability of new drugs:*	
will increase the number of patients candidate to drug therapy	236 (56.1%)
will improve the effectiveness of drug therapy	154 (36.6%)
will improve the durability of drug therapy	128 (30.4%)
will reduce the costs of treatment	53 (12.6%)
will not lead to substantial changes	32 (7.6%)
I do not know	91 (21.6%)

* more than one answer was allowed.

Orlistat was seldom prescribed (median 0; IQR 0-10), and the main reasons for the choice of this drug were represented by the failure in lifestyle changes (58.1%), failure/intolerance of other treatments for obesity (36.9%), or level of BMI (36.9%).

Similarly, naltrexone/bupropion was infrequently prescribed (median 0; IQR 0-5). Failure in lifestyle changes (59.2%), failure/intolerance of other treatments for obesity (49.0%), and level of BMI (40.8%) were reported as the major reasons for the choice of this treatment.

Finally, liraglutide was prescribed to a median of 10% of patients with obesity (IQR 10-30), and the main reasons for the choice of this drug were the presence of metabolic complications (64.3%), failure of lifestyle changes (58.7%), and level of BMI (57.4%).

Rates of prescription of the different anti-obesity drugs according to participants’ characteristics are reported in **Table 5**. Endocrinologists aged 40 years or less were more likely to prescribe metformin and liraglutide as compared to older participants. Respondents who managed obesity personally or within a dedicated team and those reporting obesity/nutrition as a major area of interest were more likely to prescribe liraglutide, orlistat, and naltrexone/bupropion.

**Table 5:** Rate of prescription of different anti-obesity drugs by respondent characteristics (median and IQR range).

Respondent characteristics	Anti-obesity drug			
	Metformin	Orlistat	Naltrexone/	Liraglutide
			Bupropion				
Age				
≤40	50 [20-60]	0 [0-2.5]	0 [0-5]	25 [10-50]
41-60	30 [10-50]	0 [0-10]	0 [0-5]	10 [0-30]
>60	25 [10-50]	3 [0-10]	0 [0-5]	5 [10-20]
p*	*0.006*	*<0.0001*	*0.8*	*<0.0001*
Gender				
Male	30 [10-50]	1 [0-10]	0 [0-10]	10 [0-30]
Female	30 [20-50]	0 [0-10]	0 [0-5]	10 [0-30]
p*	*0.07*	*0.14*	*0.08*	*0.68*
Setting of practice				
Non-University hospital	25 [10-50]	0.5 [0-10]	0 [0-5]	10 [0-30]
University hospital	37.5 [11.3-50]	0 [0-10]	0 [0-10]	20 [5-50]
Public outpatient clinic	40 [10-70]	0 [0-10]	0 [0-2]	15 [0-30]
Private outpatient clinic	35 [10-60]	0 [0-10]	0 [0-5]	10 [0-23.8]
p*	*0.053*	*0.64*	*0.21*	*0.063*
Management of obesity				
Personally/within a dedicated team	30 [20-60]	5 [0-10]	1 [0-10]	20 [5-40]
Referral to a dietician/nutritionist	30 [10-55]	0 [0-5]	0 [0-0]	10 [0-25]
Referral to a 2^nd^ level structure	20 [5-50]	0 [0-10]	0 [0-0]	9.5 [0-21.3]
p*	*0.22*	*0.002*	*<0.0001*	*<0.0001*
Main area of interest				
Obesity/nutrition	30 [10-60]	2 [0-10]	1 [0-10]	20 [5-50]
Thyroid	30 [10-50]	0 [0-10]	0 [0-1]	10 [0-25]
Diabetes	40 [10-60]	1 [0-10]	0 [0-5]	17.5 [1-37.5]
General endocrinology/other	20 [5-50]	0 [0-5]	0 [0-4]	0 [0-10]
p*	*0.28*	*0.03*	*<0.0001*	*<0.0001*

* Kruskal-Wallis one-way ANOVA.

The major obstacle to prescribing weight lowering drugs was the cost of therapy for 71.9% of participants; side effects (34.6%), limited durability of treatment (25.6%) and resistance on the part of the patient were also frequently reported barriers to anti-obesity treatment (**Table 4**).

As for expectations regarding the efficacy of interventions, half of the participants (51.4%) believed that obesity can be successfully managed in the long term with lifestyle intervention combined with drug therapy in 30% or less of the cases (**Table 4**). Among the reasons for poor adherence and lack of long-term persistence of the results of drug therapy, those more frequently reported were treatment cost (64.9%), insufficient patient motivation (55.3%), side effects (44.9%), and treatment failure (38.1%).

Psychological support was considered as an important additional component for the management of obesity. According to 41.9% of participants, psychological support should be offered to any patient with obesity, while an additional 32.2% of respondents would recommend it to half of the cases (**Table 4**).

**Table 6** reports the attitude to recommend psychological support according to respondents’ characteristics. Younger endocrinologists, female participants, and those practicing in university/non-university affiliated hospitals were more likely to recommend psychological support for all patients with obesity. On the other hand, endocrinologists who managed obesity personally or within a dedicated team, as well as those reporting obesity as a major area of practice were less likely to consider psychological support for all individuals with obesity.

**Table 6:** Reported percentage of patients to whom psychological support should be recommended by respondent characteristics.

Respondent characteristics	Percentage of patients					p*
	100%	>50%	25-50%	<25%	Only patients with psychiatric problems	
Age (years)						<0.0001
≤40	54.10%	31.50%	10.80%	2.70%	0.90%	
41-60	45.50%	34.90%	12.70%	5.30%	1.60%	
>60	26.50%	28.80%	25.00%	12.90%	6.80%	
Gender						<0.0001
Male	29.00%	34.40%	19.40%	11.80%	5.40%	
Female	51.60%	30.50%	13.40%	3.30%	1.20%	
Setting of practice						<0.0001
Non-University hospital	44.50%	31.00%	18.10%	4.50%	1.90%	
University hospital	53.20%	36.70%	7.60%	1.30%	1.30%	
Public outpatient clinic	44.00%	36.00%	12.00%	2.70%	5.30%	
Private outpatient clinic	30.80%	27.50%	20.80%	16.70%	4.20%	
Management of obesity						<0.0001
Personally/within a dedicated team	30.80%	34.10%	21.60%	8.60%	4.90%	
Referral to a dietician/nutritionist	52.80%	26.70%	13.80%	5.60%	1.00%	
Referral to a 2^nd^ level structure	46.40%	46.40%	0.00%	3.60%	3.60%
Main area of interest						0.75
Obesity/nutrition	34.10%	34.10%	19.60%	8.70%	3.60%	
Thyroid	43.90%	32.10%	14.30%	6.60%	3.10%	
Diabetes	50.00%	27.80%	13.00%	5.60%	3.70%	
General endocrinology/other	47.70%	31.80%	15.90%	4.50%	0.00%	

* Chi-square test.

Finally, only 40.5% of participants were aware of new drugs coming for obesity treatment (**Table 4**). More than half of the respondents (56.1%) believed that new drugs will increase the number of patients susceptible to therapy, while one in three was confident that new treatment options will improve the effectiveness and durability of therapy.

### Correlates of attitudes to prescribe anti-obesity drugs: Results of multivariate analyses

At multivariate analysis, the only independent correlate of the attitude to prescribe metformin was the setting of practice (**Table 7**). Endocrinologists practicing in private outpatient clinics were significantly more likely to prescribe metformin as compared to those practicing in public hospitals (OR=2.42; 95%CI 1.23-4.81).

**Table 7:** Correlates of attitudes to prescribe anti-obesity drugs: results of multivariate analysis (logistic regression with stepwise variable selection).

Respondents’ characteristics	OR	95%CI	p
Metformin			
Setting of practice			
Non-University hospital	r.c.	–	–
University hospital	1.48	0.76-2.90	0.25
Public outpatient clinic	0.95	0.52-1.75	0.87
Private outpatient clinic	2.43	1.23-4.81	0.01
Orlistat			
Age			
≤40	r.c.	–	–
41-60	1.85	1.11-3.08	0.018
>60	4.69	2.53-8.71	<0.0001
Main area of interest			
Obesity/nutrition	r.c.	–	–
Thyroid	0.74	0.45-1.24	0.25
Diabetes	1.45	0.73-2.87	0.29
General endocrinology/other	0.33	0.15-0.77	0.01
Management of obesity			
Personally/within a dedicated team	r.c.	–	–
Referral to a dietician/nutritionist	0.48	0.30-0.78	0.003
Referral to a 2^nd^ level structure	0.39	0.16-0.95	0.04
Setting of practice			
Non-University hospital	r.c.	–	–
University hospital	1.07	0.60-1.90	0.82
Public outpatient clinic	0.57	0.32-1.00	0.05
Private outpatient clinic	0.51	0.29-0.89	0.02
Naltrexone/Bupropion			
Main area of interest			
Obesity/nutrition	r.c.	–	–
Thyroid	0.5	0.30-0.82	0.007
Diabetes	0.92	0.47-1.80	0.8
General endocrinology/other	0.49	0.22-1.11	0.09
Management of obesity			
Personally/within a dedicated team	r.c.	–	–
Referral to a dietician/nutritionist	0.37	0.23-0.61	<0.0001
Referral to a 2^nd^ level structure	0.22	0.07-0.65	0.007
Liraglutide			
Main area of interest			
Obesity/nutrition	r.c.	–	–
Thyroid	0.52	0.32-0.85	0.01
Diabetes	0.64	0.32-1.24	0.19
General endocrinology/other	0.14	0.07-0.28	<0.0001

r.c. Reference category.

The attitude to prescribe orlistat was associated with respondents’ age. Endocrinologists aged 41-60 years (OR=1.85; 95%CI 1.11-3.08) and to a larger extent those aged over 65 (OR=4.69; 95%CI 2.53-8.71) were more likely to prescribe orlistat as compared to younger participants. Orlistat was less likely to be prescribed by endocrinologists with main interest in general endocrinology as compared to those interested in obesity/nutrition (OR=0.33; 95%CI 0.15-0.77). Participants who tended to refer their patients with obesity to a dietitian/nutritionist (OR=0.48; 95%CI 0.30-0.78) and those who referred patients to a 2^nd^ level structure (OR=0.39; 95%CI 0.16-0.95) were significantly less likely to prescribe orlistat than those managing personally these patients. Endocrinologists practicing in private outpatient clinics also showed a lower likelihood of prescribing orlistat as compared to those practicing in public hospitals (OR=0.51; 95%CI 0.29-0.89).

Naltrexone/Bupropion was less likely to be prescribed by endocrinologists with main interest in thyroid diseases as compared to those interested in obesity/nutrition (OR=0.50; 95%CI 0.30-0.82). Also, endocrinologists who tended to refer their patients with obesity to a dietitian/nutritionist (OR=0.37; 95%CI 0.23-0.61) and those who referred patients to a 2^nd^ level structure (OR=0.22; 95%CI 0.07-0.65) were significantly less likely to prescribe naltrexone/bupropion as compared to those managing personally these patients.

Finally, participants with a main interest in thyroid diseases (OR=0.52; 95%CI 0.32-0.85) and those with a main interest in general endocrinology (OR=0.14; 95%CI 0.07-0.28) were significantly less likely to prescribe liraglutide as compared to endocrinologists interested in obesity/nutrition.

## Discussion

The results of this large-scale survey demonstrate that obesity is widely impacting on the clinical activity of Italian endocrinologists. Over one third of the participants referred that from one in three to more than half of the patients they see in their practice have obesity. However, one in five respondents addressed the problem of obesity in 25% or less of the patients seeking care for another endocrinological problem. Younger endocrinologists were less likely to deal with obesity in most of their patients, probably as a consequence of limited experience in the management of the condition. Similarly, endocrinologists practicing in a hospital were less likely than those practicing in outpatient clinics to address obesity in the majority of their patients, possibly for the difficulties in ensuring long-term follow-up in the hospital setting. Of note, participants who reported to manage obesity personally were also more likely to deal with the problem in most of their patients. However, less than half of the participants managed obesity personally or within a dedicated team, while the remainders tended to refer their patients to dietologists/nutritionists or to structures specialized in the treatment of obesity.

Anti-obesity drugs were considered as the preferred initial approach by 65.2%, 38.5% and 12.6% of respondents in patients with obesity class 1, class 2 and class 3, respectively. The proportion of endocrinologists referring their patients to a specialized center increased with BMI; on the other hand, even in the presence of severe obesity, only one third of participants considered bariatric surgery as the first option. These results underline the importance of organizing the care of the person with obesity within shared diagnostic-therapeutic paths, in the context of a multidisciplinary approach.

The survey also shows that anti-obesity drugs are largely underutilized in real world clinical practice. The drug most commonly used is metformin, mainly prescribed in the presence of hyperinsulinemia and metabolic complications. However, while metformin represents a well-tolerated, safe treatment, its efficacy in reducing body weight is modest (19), and the drug does not have marketing authorization for the treatment of obesity in Italy. Among the authorized drugs, the most commonly utilized is liraglutide, which was however prescribed by participants to a median of 10% of their patients with obesity. Very low percentages of use of orlistat and naltrexone/bupropion were reported, mainly prescribed after failure of lifestyle changes or other drug therapy for obesity. The attitude to prescribe anti-obesity drugs was influenced by respondents’ characteristics. Younger endocrinologists were more likely to prescribe metformin and liraglutide, while those who managed obesity personally and those reporting obesity/nutrition as a main area of interest were more likely to prescribe liraglutide and, albeit in a limited number of patients, orlistat and naltrexone/bupropion. The lower attitude of endocrinologists with older age to prescribe pharmacological treatment could depend at least in part on the past negative experience with other weight lowering drugs such as sibutramine and rimonabant, withdrawn due to adverse events (cardiovascular diseases and psychological events, respectively) (20, 21). Multivariate analysis showed that the attitude of diabetologists to prescribe anti-obesity medication did not differ compared to endocrinologists dealing with obesity; this is probably due to the awareness of diabetologists about the importance of weight control in patients with diabetes. Furthermore, diabetologists, have a greater confidence in prescribing anti-obesity medications, especially GPL1 RAs, and have a better understanding of side effects and their management. The low rate of use of pharmacological treatment is in contrast with the expectations of participants. In fact, nearly half of the respondents believed that over 30% of people with obesity can be successfully managed in the long term with lifestyle intervention combined with drug therapy.

Among the barriers to prescription of anti-obesity drugs, the cost of treatment was considered a major problem by 71.9% of participants, and this factor was deemed to impact unfavorably on the adherence to long-term treatment. In this respect, it should be underlined that the Italian National Health System does not reimburse the cost of treatment, thus posing a serious obstacle to a long-lasting therapy. Among the other barriers to drug therapy, side effects and limited durability/poor effectiveness were frequently mentioned. The perception that currently available weight loss medications could be not safe or effective confirms previous findings (13, 22) and can at least partially explain the low rate of prescription of these drugs. One in five participants mentioned resistance on the part of the patient as an obstacle to prescribing anti-obesity drugs, and one in two listed insufficient patient motivation among the reasons for poor adherence and long-term persistence of the results of drug therapy. Several factors can contribute to the poor acceptance of therapy by the patient, including the cost of therapy, the occurrence of side effects, or the limited efficacy. Furthermore, poor acceptance of obesity as a chronic condition, the difficulties in viewing weight loss treatment as a long-term, rather than a short-term solution, factors within a patient’s social environment that can undermine weight-loss attempts, or the presence of a multitude of comorbidities can contribute to the resistance on the part of the patient (23). Endocrinologists should thus be trained to the early identification of treatment barriers to increase the prospect of long-term success.

Psychological support represents an important component of the behavioral intervention for people with obesity, to enhance adherence to prescriptions for a reduced-calorie meal plan, increased physical activity, and pharmacological treatment (7). Furthermore, psychologists and psychiatrists should be involved in the initial diagnostic workup and in the treatment of eating disorders, depression, anxiety, psychoses, and other psychological problems that frequently impair the effectiveness of lifestyle intervention programs. In our survey, 41.9% of participants would recommend psychological support for all people with obesity, and an additional 32.2% would recommend it to over half of the cases. The attitude to recommend psychological support was strongly influenced by participants’ characteristics. In fact, female endocrinologists and younger ones were significantly more likely to consider psychological support to all people with obesity. On the other hand, physicians practicing in private outpatient clinics, those managing obesity personally or within a dedicated team and those reporting obesity/nutrition as a major area of interest were less likely to consider psychological support for all people with obesity.

Finally, over half of the participants were not aware of new drugs expected to be available in the near future. However, there were high expectations that new drugs could increase the number of patients candidate to weight lowering therapy, and that novel therapies will improve treatment effectiveness and durability.

Our findings are supported by previous research in the field. In particular, the Awareness, Care, and Treatment In Obesity maNagement-International Observation (ACTION–IO) study was conducted to identify the perceptions, attitudes, behaviors and barriers to effective obesity care across people with obesity (PwO) and physicians (24). The ACTION IO study collected data by an online survey in Australia, Chile, Israel, Italy, Japan, Mexico, Saudi Arabia, South Korea, Spain, the United Kingdom (UK), and the United Arab Emirates (UAE).

Overall, data from ACTION IO suggest that PwO are motivated to lose weight and that there is an opportunity for physicians to initiate earlier, effective weight loss. PwO may not recognize the need to reduce excess weight until it has an impact on their health, further supporting the requirement for HCPs to raise the topic of weight before such obesity‐related complications occur. The study also reveals a global need for improved education of both PwO and HCPs concerning the biological basis and clinical management of obesity. The results from the ACTION IO survey are very similar to that of the present study; we can conclude, therefore, that there is a global need for educations of physicians dealing with obesity with the aim to promote early treatment.

Our study has limitations. First, the response rate was 24.1%. However, other surveys have reported response rates ranging between 10.8% and 34% (14, 24–27). Furthermore, the sample of participants in the survey could be non-representative of the endocrinologists’ community. In this respect, sex, age, regional distribution, and professional experience of respondents were not different from the overall figures of the Italian Association of Clinical Endocrinologists members. Finally, respondents could be those more interested in obesity management. Therefore, our survey can provide an optimistic picture regarding obesity-related attitudes and practices of Italian endocrinologists.

In conclusion, our survey shows that endocrinologists regularly encounter patients with obesity, yet they often do not address the problem or manage it. Since people with obesity may not recognize the need to reduce excess weight until it has an impact on their health, it is of paramount importance for endocrinologists to raise the topic of weight for a timely intervention aimed at prevention of obesity-related complications, before obesity progresses. Currently available anti-obesity medications are safe and effective and other are coming more and more efficacious. Nevertheless, pharmacological treatment is seldom prescribed by most participants, and several barriers to effective obesity management are perceived. It is therefore of primary importance to address the unmet educational needs of endocrinologists to raise their awareness towards the problem of obesity and to increase their confidence in managing this pathological condition with a pervasive impact on our society.

## Data availability statement

The raw data supporting the conclusions of this article will be made available by the authors, without undue reservation.

## Author contributions

All authors contributed to the study conception and design. Data analysis was performed by AN. The first draft of the manuscript was written by AN and all authors commented on previous versions of the manuscript. All authors contributed to the article and approved the submitted version.
